# Retraction Note: Tumour microenvironment programming by an RNA–RNA-binding protein complex creates a druggable vulnerability in IDH-wild-type glioblastoma

**DOI:** 10.1038/s41556-025-01613-0

**Published:** 2025-01-16

**Authors:** Lele Wu, Zheng Zhao, Yong Jae Shin, Yiyun Yin, Anandhkumar Raju, Thamil Selvan Vaiyapuri, Khaireen Idzham, Miseol Son, Yeri Lee, Jason K. Sa, Joelle Yi Heng Chua, Bilal Unal, You Zhai, Wenhua Fan, Lijie Huang, Huimin Hu, Jayantha Gunaratne, Do-Hyun Nam, Tao Jiang, Vinay Tergaonkar

**Affiliations:** 1 Laboratory of NFκB Signalling, Institute of Molecular and Cell Biology (IMCB), Agency for Science Technology and Research (A*STAR), Singapore, Republic of Singapore; 2 Beijing Neurosurgical Institute, Capital Medical University, Beijing, China; 3 Research Institute for Future Medicine, Samsung Medical Center, Seoul, Republic of Korea; 4 Department of Biomedical Sciences, Korea University College of Medicine, Seoul, Republic of Korea; 5 Beijing Tiantan Hospital, Capital Medical University, Beijing, China; 6 Laboratory of Translational Biomedical Proteomics, Institute of Molecular and Cell Biology (IMCB), Agency for Science Technology and Research (A*STAR), Singapore, Republic of Singapore; 7 Department of Neurosurgery, Samsung Medical Center, Seoul, Republic of Korea; 8 Department of Pathology, Yong Loo Lin School of Medicine, National University of Singapore (NUS), Singapore, Republic of Singapore; 9 Department of Biochemistry, Yong Loo Lin School of Medicine, National University of Singapore (NUS), Singapore, Republic of Singapore

Retraction to: *Nature Cell Biology* (2024) 26:1003-1018 10.1038/s41556-024-01428-5

The authors are retracting this Article after concerns over several image irregularities were brought to our attention, including but not limited to the following:

- In Fig. 1f, there appears to be a partial overlap between the Control siRNA and WWTR1-AS1 siRNA panels;
- In Fig. 6b, the WT-KO and KO-WT mice appear very similar;
- In Fig. 6d, there appears to be a partial overlap between the WT-KO and KO-WT panels;
- In Fig. 7a, the leftmost images under GMB192 and GMB925 appear very similar;
- In Fig 7d, there appears to be partial overlap between all four panels;
- In Fig. 7f, panels 4 and 6 in Week 3 - DHX inhibitor + TMZ appear very similar;
- In Extended Data Fig. 6h, the images under DMSO and DHX inhibitor appear very similar;
- In Extended Data Fig. 8d panel PE-A and Extended Data Fig 8e, panel WT-KO, appear very similar.

The authors attribute image overlap and similarities to human error. Given the number of errors, however, they have lost confidence in the reliability of the study and therefore wish to retract the Article. The authors deeply regret these errors and sincerely apologise to the community. All authors agree to this retraction.

